# P-1300. Themes to Support Identification of Emerging Infections Diseases

**DOI:** 10.1093/ofid/ofae631.1481

**Published:** 2025-01-29

**Authors:** Andrea F Kalvesmaki, Kelly Peterson, Christian Dalton, Joann Vuong, Barbara E Jones, Vanessa W Stevens, Makoto Jones

**Affiliations:** VA Salt Lake City Healthcare System, Salt Lake City, Utah; Veterans Affairs, Veterans Health Administration, Office of Analytics and Performance Integration; University of Utah, Division of Epidemiology, Salt Lake City, Utah; University of Utah, Salt Lake City, Utah; University of Utah, Veterans Affairs, Salt Lake City, Utah; University of Utah and Salt Lake City VA Healthcare System, Salt Lake City, Utah; US Department of Veterans Affairs, Salt Lake City, Utah; University of Utah, VA Salt Lake City Health Care System, Salt Lake City, Utah

## Abstract

**Background:**

Many outbreaks are first recognized by astute clinicians who notice novel disease presentations, even before statistical anomalies are detected . In our efforts to identify COVID-19 cases using automated surveillance, we noted that the tone and content of clinical notes written early in the pandemic were much different than those written later . We investigated the content of early outbreak notes to identify general patterns that might reflect an emerging disease.

**Methods:**

This study seeks to identify themes in a sample of clinical notes from Veteran Administration (VA) data. We used a convenience sample of early COVID-19 , early mpox, and CDC Nationally Notifiable Diseases cases as well as inference from a natural language processing (NLP) classifier that the clinical document mentioned communication with a public health authority (as an indicator of concern). These notes were reviewed by a VA infectious disease expert and a VA medical anthropologist with training in global health, the medical explanatory model, and diagnostic decision-making. Thematic analysis was applied to the notes.

**Results:**

Five main themes were detected in the 100 documents reviewed. 1-Provider discerned something abnormal and included terms such as “unusual presentation”, and “disproportionate” to describe specific symptoms and indicate an element of being surprised. 2-Initiation of protocols for containment or treatment included terms such as “isolate” and “out of concern for… will treat with ” followed by a description of an established protocol for one or more symptoms. 3-Justification of protocol deviation included terms like “insist(ing)” on a type of test or referral to the health authorities. 4-Exposures included terms detailing locations of travel, potentially exposed persons, or locations that may have been sites of initial contraction. 5-Patient voice, as a theme, were described patient fear or agitation.

**Conclusion:**

Unusual cases can prompt documentation of their exceptionality. Similar patterns may be seen in emerging diseases, such as H5N1, perhaps well before statistical increases are detected in the syndromes they cause. In further research, we will expand on this limited sample and explore NLP systems that could be applied to health records to scan for emerging health threats.

**Disclosures:**

**All Authors**: No reported disclosures

